# The protective role of transferrin in Müller glial cells after iron-induced toxicity

**Published:** 2008-05-20

**Authors:** Emilie Picard, Isabelle Fontaine, Laurent Jonet, Florian Guillou, Francine Behar-Cohen, Yves Courtois, Jean-Claude Jeanny

**Affiliations:** 1 Inserm, U872, Paris, France; 2 Centre de Recherche des Cordeliers, Université Pierre et Marie Curie, Paris, France; 3 Université Paris Descartes, Paris, France; 4 UMR 6175, Institut National de la Recherche Agronomique, Centre National de Recherche Scientifique, Université de Tours, Haras Nationaux Physiologie de la Reproduction et des Comportements, Nouzilly, France

## Abstract

**Purpose:**

Transferrin (Tf) expression is enhanced by aging and inflammation in humans. We investigated the role of transferrin in glial protection.

**Methods:**

We generated transgenic mice (Tg) carrying the complete human transferrin gene on a C57Bl/6J genetic background. We studied human (hTf) and mouse (mTf) transferrin localization in Tg and wild-type (WT) C57Bl/6J mice using immunochemistry with specific antibodies. Müller glial (MG) cells were cultured from explants and characterized using cellular retinaldehyde binding protein (CRALBP) and vimentin antibodies. They were further subcultured for study. We incubated cells with FeCl_3_-nitrilotriacetate to test for the iron-induced stress response; viability was determined by direct counting and measurement of lactate dehydrogenase (LDH) activity. Tf expression was determined by reverse transcriptase-quantitative PCR with human- or mouse-specific probes. hTf and mTf in the medium were assayed by ELISA or radioimmunoassay (RIA), respectively.

**Results:**

mTf was mainly localized in retinal pigment epithelium and ganglion cell layers in retina sections of both mouse lines. hTf was abundant in MG cells. The distribution of mTf and hTf mRNA was consistent with these findings. mTf and hTf were secreted into the medium of MG cell primary cultures. Cells from Tg mice secreted hTf at a particularly high level. However, both WT and Tg cell cultures lose their ability to secrete Tf after a few passages. Tg MG cells secreting hTf were more resistant to iron-induced stress toxicity than those no longer secreted hTf. Similarly, exogenous human apo-Tf, but not human holo-Tf, conferred resistance to iron-induced stress on MG cells from WT mice.

**Conclusions:**

hTf localization in MG cells from Tg mice was reminiscent of that reported for aged human retina and age-related macular degeneration, both conditions associated with iron deposition. The role of hTf in protection against toxicity in Tg MG cells probably involves an adaptive mechanism developed in neural retina to control iron-induced stress.

## Introduction

All cells require iron for survival and as a cofactor for a variety of enzymes [1]. However, iron is also highly toxic due to its ability to generate free radicals via the Fenton reaction. Homeostatic mechanisms maintain iron levels by regulation of the proteins involved in iron import, storage, and export, thus preventing deleterious consequences of either iron overload or deficiency.

Diseases such as aceruloplasminemia and age-related macular degeneration (AMD) and conditions such as siderosis bulbi and subretinal hemorrhage are associated with increased intraocular iron levels, contributing to subsequent retinal degeneration [2–7]. The eye is particularly iron-dependent. For instance, the extensive membrane biogenesis necessary to replenish shed photoreceptor outer segments (OS) requires iron as an essential cofactor [8]. Isomerization of all-trans-retinal within the retinal pigment epithelium (RPE) in the visual cycle requires iron for activation of RPE65, an enzyme involved in the visual cycle pathway [9]. Moreover, being part of the blood-retinal barrier, the RPE regulates the flow of iron and other nutrients between the choroidal vasculature and the outer retina. The highest levels of iron are found in the choroid, RPE, and photoreceptor inner segments (IS) and OS in normal adult rat and human retina [10–12]. Accumulation of iron has been observed in Royal College of Surgeons (RCS) rats, in which phagocytosis of OS by RPE is impaired [13], and in ceruloplasmin and hephaestin double knockout mice [14]. The study of the roles of iron-handling proteins in the retina will allow the normal mechanisms of iron detoxification to be understood.

The major pathway for iron import involves the binding of transferrin (Tf) to its receptor on the cell surface and subsequent endocytosis. Tf is an extracellular protein, binding and transferring iron within and across tissues. It is a ubiquitous protein with a crucial role in iron transport and iron homeostasis [15,16]. This protein is found in the biologic fluids of vertebrates, mainly synthesized in hepatocytes [17], and secreted into the blood. In adults, Tf synthesis is also found in other cell types including oligodendrocytes [18], choroid plexus epithelial [19], Sertoli [20], mammary epithelial [21], and retinal cells (including RPE) [10]. Tf may protect the retina from the potentially toxic effects of unbound iron; indeed, iron bound to Tf does not cause oxidative stress. Finally, Tf seems to be essential for proliferation-promoting activity of non-neuronal cells and survival of neuronal cells [22,23].

We used a transgenic mouse (Tg) carrying the complete human transferrin (hTf) gene to study the protective effect of Tf against oxidative stress. In Tg mice, hTf mRNA has been found in hepatocytes, oligodendrocytes, and Sertoli cells of the testis [24]. In testis and brain, mouse Tf (mTf) is upregulated when compared to control mice. It has been shown that myelination and maturation of oligodendrocytes in Tg mice induce behavioral improvements [25,26]. Sixteen-month-old Tg mice have low testicular sperm reserves without consequence on fertility [27].

In the present work, we first examined the retinal distribution of hTf and mTf in Tg mice and found that hTf is produced in RPE and Müller glial (MG) cells. Second, we isolated and cultured MG cells from Tg and wild-type (WT) mice and examined the potential role of hTf over-production in protecting Tg MG cells exposed to FeCl_3_-nitrilotriacetate (FeCl_3_-NTA). Finally, we compared its action to the effects of exogenous human apo-Tf and holo-Tf on MG cells from WT mice.

## Methods

### Animals

Tg mice carrying the long hTf gene (80 kb) comprising its long 5′- and 3′-regulatory sequences [24] were generated from the 803 line previously described [26] backcrossed in the C57Bl/6J background. They were screened for the presence of hTf in the blood, using ELISA. All Tg mice used in this study were homozygous for the hTf gene. Control animals were WT C57Bl/6J mice (Janvier, Le Genest, St. Isle, France). All mice were fed a standard laboratory diet and tap water ad libitum and maintained in a temperature controlled room at 21–23 °C with a 12h:12h light-dark photoperiod. Experimental procedures were performed in accordance with the Association for Research in Vision and Ophthalmology (ARVO) statement for the use of animals in ophthalmology and vision research and were approved by each author's institutional animal care review board.

**Table 1:** Overview of all primers used in the reverse transcriptase polymerase chain reaction.

**Gene name**	**Forward primer (5′-3′)**	**Reverse primer (5′-3′)**	**References**
Mouse transferrin	GGACGCCATGACTTTGGATG	GCCATGACAGGCACTAGACC	[48]
Human transferrin	CCCTTAACCAATACTTCGGCTAC	GCCAAGTTCTCAAATATAGCTGAG	[59]
Mouse β-actin	GACGGCCAAGTCATCACTATTG	CCACAGGATTCCATACCCAAGA	[60]

Forward and reverse primers sequences used to detect mouse and human transferrin and mouse beta actin genes with the reverse transcriptase polymerase chain reaction method.

### Immunohistofluorescence analysis

Freshly enucleated eyes from one-month-old WT and Tg mice were fixed by incubation in 4% paraformaldehyde (Inland Europe, Conflans sur Lanterne, France) in 1X phosphate-buffered saline (PBS, Gibco distributed by Invitrogen, Cergy Pontoise, France) for 2 h, then mounted in Tissue Tek O.C.T. (Siemens Medical, Puteaux, France) and frozen with dry ice. Next, 10 µm frozen sections were cut on a Leica CM3050S freezing microtome (Leica, Rueil Malmaison, France). Sections were incubated for 1 h with primary antibodies diluted in PBS, as follows: 1:100 rabbit polyclonal anti-mTf (Florian Guillou, INRA, France) [28], 1:100 rabbit polyclonal anti-hTf (Florian Guillou) [29], and 1:250 rabbit anticellular retinaldehyde binding protein (CRALBP; received as a gift from John Saari, University of Washington, Seattle, WA). Control sections were incubated with non-immune rabbit serum (Invitrogen, Cergy Pontoise, France). For CRALBP antibody, sections were first blocked with 1% BSA (Sigma, Saint Quentin Fallavier, France) and 10% normal goat serum (Invitrogen) before incubation with the primary antibody. An eye from a 90-year-old man (gift from Monica Valtink, Uni-Augenklinik Dresden, University Eye Hospital, Dresden, Germany) was mounted, cut, and examined using the aforedescribed procedure, for the presence of hTf.

Sections were washed with PBS and then incubated for 1 h with secondary antibodies (goat antirabbit) labeled with Texas Red (Jackson Laboratories distributed by TebuBio, Le Perray en Yvelines, France), Alexa 596, or Alexa 488 (Invitrogen).

Nuclei were counterstained with 4’,6-diamidino-2-phenyl-indole (Sigma). Sections were analyzed by fluorescence microscopy using a Leica Aristoplan and photographed with a Spot camera (Optilas, Evry, France), using identical exposure parameters across samples to be compared.

### Preparation of retinal extracts

Retinal extracts were obtained by incubating 5 mg retinal tissue, obtained from one-month-old WT and Tg mice in lysis buffer. The buffer was composed of 15 mM Tris, pH 7.9, 60 mM KCl, 15 mM NaCl, 2 mM EDTA, and 0.4 mM phenylmethylsulphonyl fluoride (Perbio Science, Brebiers, France). After four successive freeze–thaw cycles (liquid nitrogen-room temperature), lysates were centrifuged at 5,000x g for 10 min, and supernatants were stored at –20 °C. hTf and mTf were measured in equal volumes of supernatant by ELISA and radioimmunoassay (RIA), respectively. Results were reported in ng/ml of supernatant.

### Transferrin quantification

hTf and mTf from retinal extracts of WT and Tg mice were quantified by an antibody-sandwich ELISA, as previously described [29]. The microtitration plate was coated with rabbit anti-hTf (Florian Guillou), then different concentrations of hTf (Sigma) or samples to be quantified were added. The plate was incubated with sheep anti-hTf (Invitrogen) and with a peroxydase-conjugated-anti-sheep-immunoglobin (Sigma). Enzyme activity was determined using alpha-phenylenediamine (Sigma) as substrate and absorbance was read at 492 nm. The minimun limit for detection was 0.1 ng/ml. The cross-reaction rate for hTf antibodies with mTf was less than 0.05%. mTf was measured by RIA, as previously described [28]. RIA was set up using ^125^I-labeled-mTf. The minimun limit for detection was 1 ng/ml. The cross-reaction rate for mTf antibodies with hTf was less than 0.0001%. All standards and samples were assayed in triplicate. The cross-reaction rate for mTf antibodies with hTf was less than 0.0001%.

### Human transferrin and mouse transferin analysis by RT-qPCR

Eyes from one-month-old WT and Tg mice were enucleated, and retinas were completely dissected and homogenized using a pellet pestle motor (Dutscher, Issy les Moulineaux, France) in lysis buffer and frozen at −80 °C after centrifugation. Lysates were thawed and RNA isolated using an RNeasy mini kit column (Qiagen, Courtaboeuf, France). First strand cDNA was obtained by standard reverse transcriptase (RT) reaction (Invitrogen). Next, 150 ng RNA was reverse transcribed for 50 min at 42 °C with 200 U SuperScript II Reverse Transcriptase (Invitrogen) and oligod(T). One µl of Rnase H was added to each sample and incubated for 20 min at 37 °C before target cDNA amplification. A qPCR mastermix was prepared to a final volume as indicated: 7.5 µl water, 0.1 µl mixed forward and reverse primers (0.25 µM each; MWG Biotech Fr, Champlan, France), 0.4 µl Rox Reference Dye, and 10 µl Platinum SYBR Green qPCR Super-Mix-UDG (Invitrogen). Next, 18 µl aliquots were each added to 2 µl of cDNA (1:10 dilution) in a reaction plate (Applied Biosystems, Applera France SA, Courtaboeuf, France) . Please see Table 1 for detailed information about primers.

**Figure 1:**
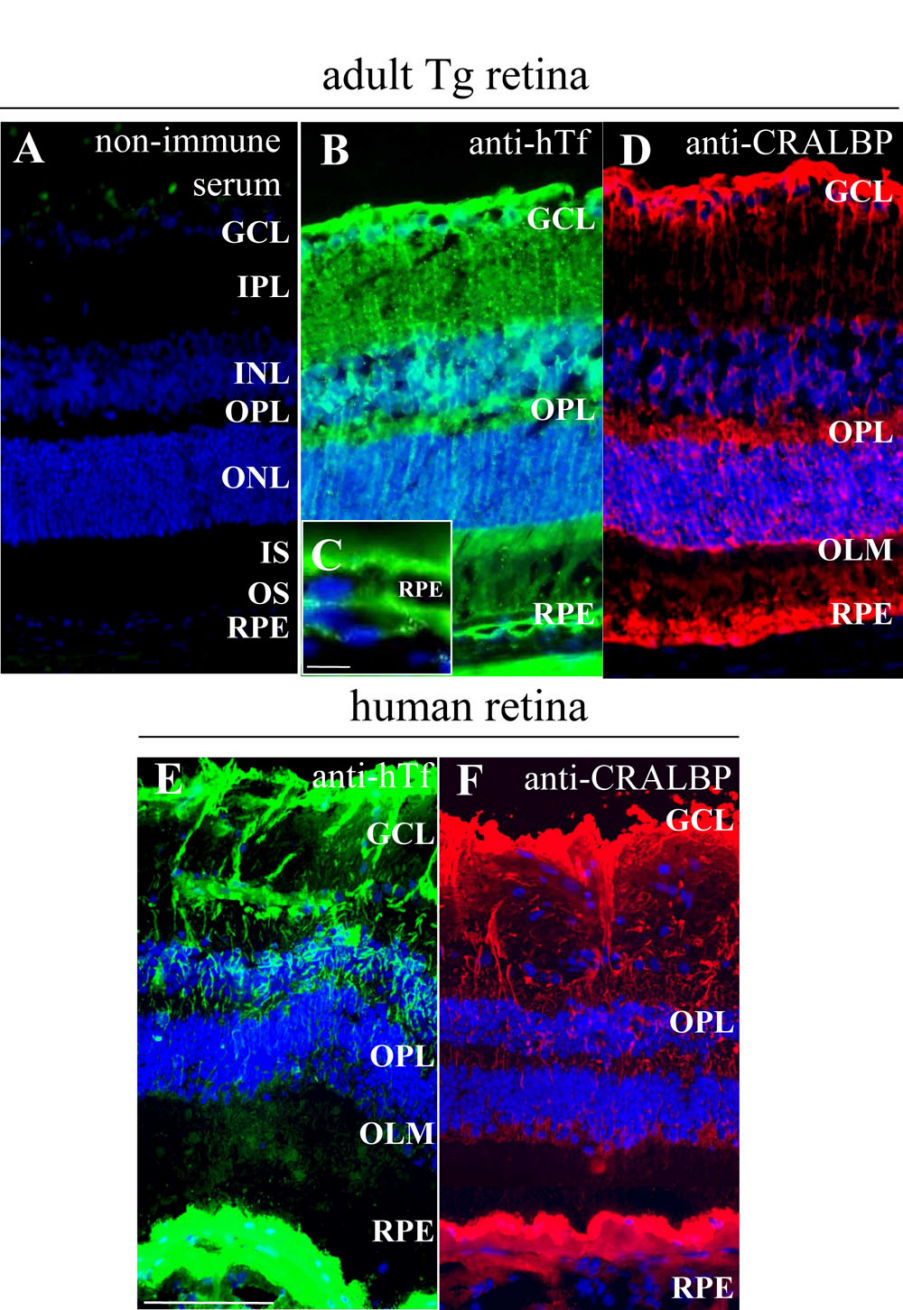
Human transferrin is localized in the same cells in transgenic mice retina as in human retina. A: Non-immune serum was used as a control. B-D: Human transferrin (hTf; green) localization was realized on frozen sections of one-month-old transgenic (Tg) mice retina. hTf is localized in Müller glial (MG) cells and retinal pigment epithelium (RPE; **B,C**), identified with cellular retinaldehyde binding protein (CRALBP; red; **D**), specific marker. C: Higher magnification of RPE from (**B**). E-F: hTf is localized in a 90-year-old human retina (**E**) in the same cells as in Tg mice still identified by CRALBP (**F**). The following abbreviations are used: Ganglion cell layer (GCL), inner nuclear layer (INL), inner plexifrom layer (IPL), inner segments (IS), outer limiting membrane (OLM), outer nuclear layer (ONL), outer plexifrom layer (OPL), outer segments (OS), retinal pigmented epithelium (RPE). The scale bars in **A**,**B**, **D-F** equal 100 µM and the scale bar in **C** equals 10 µm.

Amplification was performed using an Applied Biosystems 7300 Real-Time PCR System. The following conditions were used: 95 °C for 10 min (denaturation); 40 cycles of 95 °C for 15 s and 60 °C for 1 min (amplification and quantification); and 95 °C for 15 s, 60 °C for 30 s, and 95 °C for 15 s (melting curve). Crossing points (CP) for each transcript—the point at which the fluorescence is significantly higher than background fluorescence—were determined for subsequent analysis.

### Cell cultures

Four or five WT or Tg mice were killed between postnatal day 8 and 12 (PN8–12) by decapitation. Next, eyes were rapidly enucleated, transferred into Dulbecco’s Modified Eagle Medium (DMEM; Invitrogen), and stored overnight at room temperature in the dark. Intact globes were then incubated in DMEM containing 2 mg/ml trypsin 250 (Invitrogen) and 2 mg/ml collagenase Type I (Worthington, Coger, Paris, France) at 37 °C for 60 min. Globes were placed in a Petri dish containing DMEM supplemented with 10% fetal calf serum (FCS; Invitrogen), 1% penicillin and streptomycin (Invitrogen), 1% L-glutamine (Invitrogen), and 0.1% fungizone (Invitrogen). Retinas were removed, mechanically dissociated into small pieces with forceps, and seeded into 10 cm Petri dishes, each containing approximately between five and seven retinas. After three to four days, medium was replaced with fresh medium. From then on, medium was replaced every week. Pigmented eyes were used for all experiments to monitor potential contamination by foreign tissue, such as RPE or choroid. All cultures were maintained at 37 °C in a humidified incubator with 5% CO_2_/95% air. This is a recommended protocol for selection of MG cells.

### Enrichment of adherent cells and Müller glial cell passaging

Once MG cells reached semiconfluence (five to seven days), retinal aggregates and debris were removed by vigorously pipeting medium onto the dish. This was repeated three to five times to dislodge all aggregates. Cell cultures were split when they were confluent. Cells were rinsed with calcium-free PBS followed by a brief incubation in PBS containing 0.05% trypsin-EDTA (Invitrogen). The suspension was centrifuged at 1,000 rpm for 10 min, cell pellets were resuspended, and cells were seeded at 12x10^3^ cells/cm^2^ in fresh DMEM, supplemented with 10% FCS, 1% penicillin and streptomycin, 1% L-glutamine, and 0.1% fungizone.

**Figure 2:**
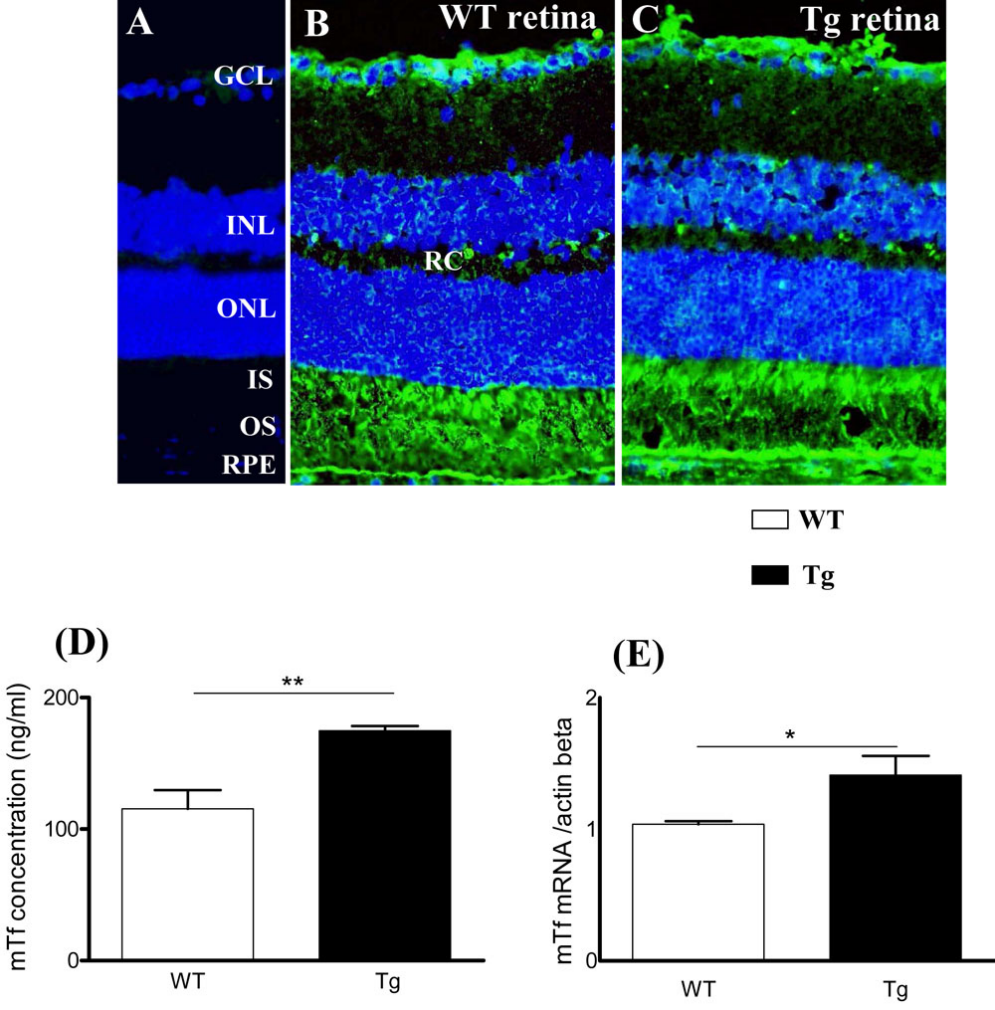
Mouse transferrin is synthetized and localized in one-month-old mouse retina. A: Non-immune serum was used as a control. B-C: Mouse transferrin (mTf; green) is localized on frozen sections of wild-type (WT) mice (**A,B**) and in transgenic (Tg) mice (**C**). Scale bar equals 100 µM. The following abbreviations are used: Ganglion cell layer (GCL), inner nuclear layer (INL), inner plexifrom layer (IPL), inner segments (IS), outer limiting membrane (OLM), outer nuclear layer (ONL), outer plexifrom layer (OPL), outer segments (OS), retinal capillaries (RC), retinal pigmented epithelium (RPE). **D:** mTf concentration (ng/ml) was measured by radioimmunoassay (RIA) in supernatant of retinal extracts of WT and Tg mice . Each column represents the mean ±SEM. The double asterisk represents statistical significance of differences from control, p<0.01. E: mTf mRNA expression was quantified by RT-qPCR in retinal extracts from WT and Tg mice. Each column represents the mean ±SEM. The asterisk represents statistical significance of differences from control, p<0.05.

### Müller glial cell immunocytochemistry

The homogeneity of cell cultures from WT and Tg mice was evaluated by immunocytochemistry with the specific MG cell marker, vimentin. Subcellular localization of mTf and hTf was determined using specific antibodies. First passage MG cells grown on LabTek chamber slides (VWR International, Strasbourg, France) were washed twice with PBS and fixed with 4% paraformaldehyde for 15 min. Cells were permeabilized with 0.1% Triton X-100 in PBS for 10 min and blocked with 1% BSA solution for 30 min. Primary antibodies; rabbit anti-mTf, rabbit polyclonal anti-hTf, and rabbit antivimentin were each diluted in PBS containing 0.5% BSA. As per the aforedescribed procedure, MG cells were treated with secondary antibodies for retinal sections.

### Exposure of cells to iron

MG cells from WT and Tg mice were incubated with 1:4 freshly prepared FeCl_3_-NTA solution (François Canonne-Hergaux, Bichat, France). NTA is a small molecular weight chelator, sequestering Fe^3+^ and forming hydrophobic complexes [30]. Cells were seeded at a density of 12x10^3^ cells/cm^2^ in 24 or 12 well plates and incubated in DMEM containing 10% FCS, 1% penicillin and streptomycin, 1% L-glutamine, and 0.1% fungizone. Cells were counted when they were confluent (after three days). Cells were washed in PBS and incubated in serum-free medium containing FeCl_3_-NTA at a final concentration of 100 µM or 500 µM for 96 h. Cells incubated in medium without FeCl_3_-NTA served as controls.

### Exogenous human transferrin treatment

MG cells from WT mice were treated with exogenous human apo and holo-Tf (Sigma). As per the aforedescribed protocol, first passage MG cells were prepared, before iron exposure. Cells were incubated for 96 h in serum-free medium containing 100 µM FeCl_3_-NTA with freshly prepared hTf solution added to final concentrations of 2.5, 5, 10, and 20 ng/ml. Incubations with the four hTf concentrations but without FeCl_3_-NTA were also performed. For quantification purposes, cell counts for cultures incubated without FeCl_3_-NTA were defined as 100%.

### Cell number evaluation

Cells were counted using a Malassez cell. After treatment, cells were removed from plates as described in the next section. Cell pellets were resuspended in DMEM containing 10% FCS. Suspension aliquots were placed in a Malassez cell, and cells were counted under an inverted microscope DMIRB (Leica) equipped with phase contrast. Results, normalized to the volume of the cell suspension, were expressed as percentages ±standard error of MG cells under control conditions (without 100 µM FeCl_3_-NTA), for WT and Tg mice.

### Determination of cell viability

Cell viability was determined by measuring lactate dehydrogenase (LDH) activity in the incubation medium with a microtiter plate assay (Nunc distributed by VWR International). Next, 100 µl aliquots of incubation medium were collected following iron stress and incubated with 100 µl of Cytotoxicity Detection Kit reaction mixture (Roche Diagnostics, Meylan, France) for 30 min at room temperature in the dark. The optical density of the solution was measured on an ELISA plate reader with 492 and 620 nm filters (Benchmark Plus microplate spectrophotometer, BioRad, Marnes-la-Coquettes, France). LDH activity in the incubation medium was compared with that measured after complete lysis of the cells in medium containing 2% Triton X-100. A viability percentage of zero corresponded to 100% LDH activity in the medium.

### Transferrin quantification and RT-qPCR analysis of human transferrin and mouse transferrin

hTf and mTf were measured by ELISA and RIA, respectively, in MG cell medium and in MG cells (from WT and Tg mice) following iron stress treatment, according to the method described for hTf and mTf retinal extract measurements.

For RT-qPCR analysis, MG cells, treated with or without iron, were lysed in buffer and lysates were frozen at −80 °C. Methods used were as described in the previous section for RT-qPCR analysis of retinal extracts.

### Statistical analysis

Results were presented as mean ±standard error of the mean. We used the Mann–Whitney U-test to evaluate differences between Tg and WT mice. Analysis was performed using GraphPad Prism 4 software. A p<0.05 was considered statistically significant. Each experimental condition was repeated three or four times, and MG cells used were from different primary cell cultures.

## Results

### Characterization of transgenic mice and detection of transferrin protein and mRNA

hTf protein was detected in Tg mouse retina by immunohistochemistry. hTf was observed in the RPE layer and MG cells identified by CRALBP immunoreactivity, a specific marker for MG cells (Figure 1B, D). hTf was present in MG cell bodies within the inner nuclear layer and in MG cell processes that form radial extensions through the retina. hTf in human retina sections had the same distribution (Figure 1E-F). These findings were confirmed by the detection of hTf protein and mRNA in Tg mouse retina by ELISA and RT-qPCR (data not shown). hTf in Tg mouse retina was detected at early stages of development (postnatal day 1) and its localization pattern correlated with MG cell differentiation during embryonic and postnatal development (data not shown).

For both WT and Tg mice, we demonstrated that mTf protein was present in the outer segments (OS) and, to a lesser extent, in the inner segments (IS) of photoreceptors, in the thin layer of RPE cells and in astrocytes of the ganglion cell layer (GCL; Figure 2B-C). mTf immunoreactivity was also detected in retinal capillaries, choroid, and sclera. Diffuse mTf immunoreactivity was evident throughout both WT and Tg mice retinas during the first days of development; mTf then became localized to the cellular layers. The patterns of mTf immunoreactivity in WT and Tg mice were similar throughout development (data not shown).

mTf protein was detected in retinal extracts from both WT and Tg mice using ELISA and RIA. However, levels of mTf were significantly higher in Tg mouse retina than in WT (Figure 2D). Consistent with these results, mTf mRNA was detected in both WT and Tg mice neural retina using RT-qPCR. mTf mRNA levels were significantly higher in Tg mouse retina than in WT retina (Figure 2E).

### Transferrin synthesis in Müller glial cells in vitro

**Figure 3:**
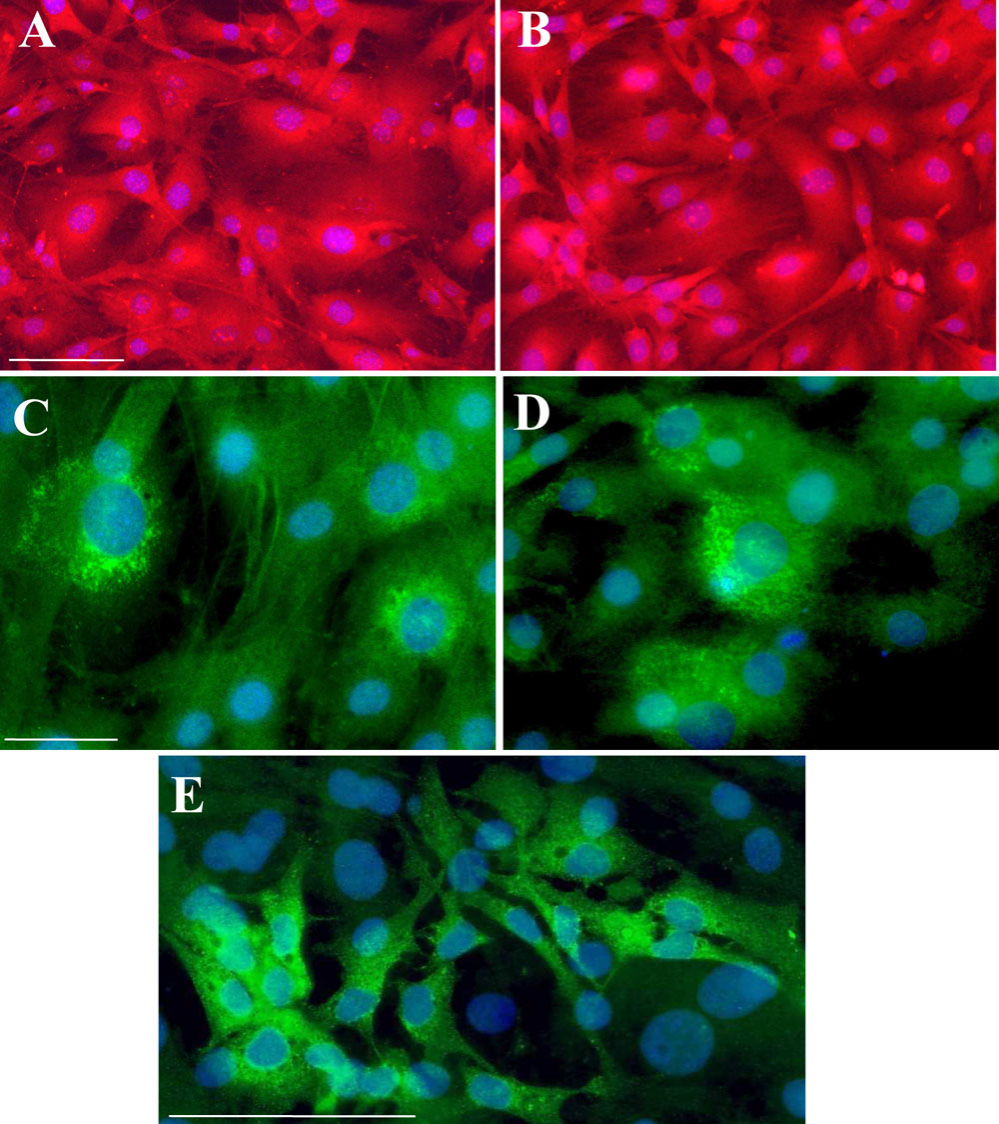
Mouse transferrin and human transferrin are present in Müller glial cells from 8- to 12-day-old wild-type and transgenic mice. A-B: Müller glial (MG) cells in culture from wild-type (WT; **A**) and transgenic (Tg; **B**) mice were identified with vimentin staining (red) specific marker. C-E: Mouse transferrin (mTf; **C,D**) and human transferrin (hTf; **E;** green) were localized in MG cells from WT (**C**) and Tg (**D,E**) mice. Scale bar: 100 µm.

To investigate the role of hTf in Tg mouse retina, we isolated MG cells from PN8–12 WT and Tg mice and cultured them as described in methods [31]. All cells from both mouse types were vimentin immunoreactive (vimentin is a specific marker of MG cells) in primary cultures (Figure 3A,B) as well as in subcultures (results not shown).

mTf and hTf protein was detected by immunocytochemistry in MG cells from WT and Tg mice. Both mTf and hTf were detected in the cytoplasm of MG cells (Figure 3C-E). However, even in primary cultures, there was some heterogeneity in the levels of protein present.

**Figure 4:**
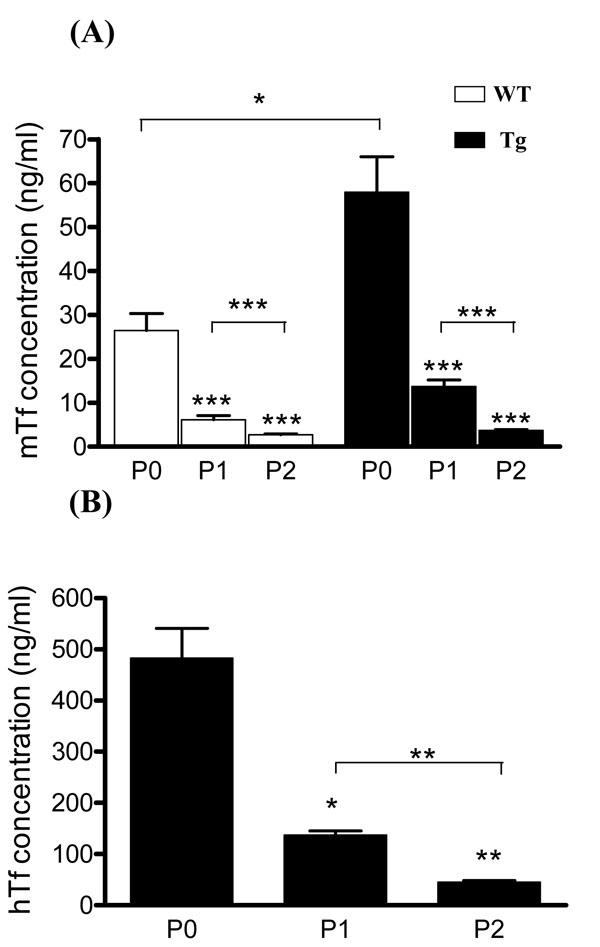
Transferrin secretion in medium of Müller glial cells from 8 to 12-day-old wild-type and transgenic mice decreases with the subcultures. A: Mouse transferrin (mTf) secretion in medium of cultured Müller glial (MG) cells from wild-type ( WT) and transgenic (Tg) mice at confluency after primary culture (P0) and at the first (P1) and the second passages (P2) was measured by radioimmunoassay (RIA; ng/ml). Each column represents the mean ±SEM. The asterisk represents statistical significance of differences between WT and Tg at P0, p<0.05. The triple asterisk represent statistical significance of differences from WT and Tg mice betwen P1 and P2 compared to P0 and between P1 and P2 themselves, p<0.001. B: Human transferrin (hTf) secretion in medium of cultured MG cells from WT and Tg mice at confluency after primary culture (P0) and at the first (P1) and the second passages (P2) was measured by ELISA assay (ng/ml). Each column represents the mean ±SEM. The asterisk represents statistical significance of differences from P0, p<0.05. The double asterisk represents statistical significance of differences from WT and Tg mice betwen P1 and P2 compared to P0 and between P1 and P2 themselves, p<0.0.1.

Media from primary cultures and subsequent passages of WT and Tg MG cells were assayed for hTf and mTf by ELISA and RIA, respectively. mTf concentration in Tg mice-derived primary culture medium was significantly twice that in WT mice-derived cell medium (Figure 4A). The concentration of hTf in MG cells from Tg mice was 10 times the mTf concentration (Figure 4B). In WT and Tg mice-derived MG cells, mTf and hTf concentrations significantly decreased progressively with the number of passages, such that Tf levels were undetectable by the third passage.

### Effects of iron stress on Müller glial cell viability in culture

**Figure 5:**
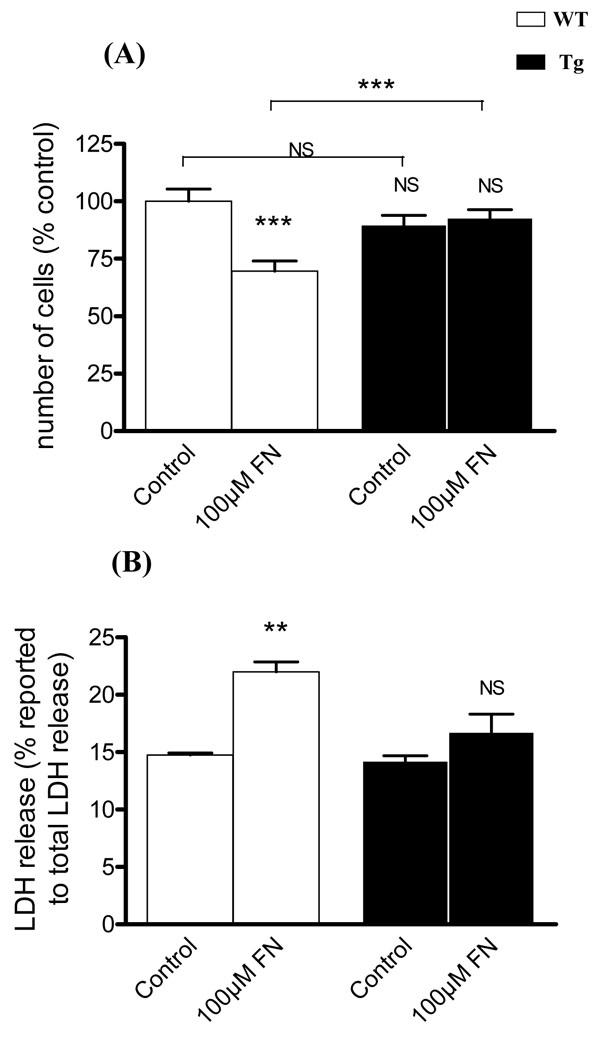
Müller glial cells from transgenic mice are more resistant to iron-mediated stress than Müller glial cells from wild-type mice. A: Culture of Müller glial (MG) cells from wild-type (WT) and transgenic (Tg) mice at the first passage were treated with 100 µM of FeCl_3_-NTA (FN), during 96 h. The number of cells was evaluated by counting in comparison to control condition. Each column represents the mean ±SEM. The triple asterisk represent statistical significance of differences between treated and control, respectively, for WT and Tg, and between control and treated, p<0.001. **B:** Release of lactate dehydrogenase (LDH) was measured after incubation of MG cells with 100 µM of FN. The percent (%) release of LDH from MG cells was reported to the total LDH release. Each column represents the mean ±SEM. The double asterisk represents statistical significance of differences from control, p<0.01 In both figures, data considered not significant is marked NS.

To test the response of MG cells from WT and Tg mice to a metabolic stress, MG cell cultures (passages one and three) were treated with two concentrations of 1:4 FeCl_3_-NTA (100 and 500 µM) in serum-free medium. The number of MG cells in first passage WT cultures treated with 100 µM FeCl_3_-NTA was 35% lower (p<0.0007) than in untreated WT cultures. Conversely, cell counts in Tg cultures treated with this same concentration of FeCl_3_-NTA were not significantly different from those in untreated cultures (Figure 5A). However, cell counts in cultures treated with 500 µM of FeCl_3_-NTA were less than 50% of those in untreated cultures (data not shown). Third passage cell cultures, with barely detectable hTf and mTf levels, treated with 100 µM FeCl_3_-NTA, had a 60% lower survival rate than untreated third passage cultures (data not shown).

LDH activity was assayed in the medium of FeCl_3_-NTA-treated, first passage WT MG cells to evaluate cell death. LDH activity was 8.6% significantly higher (p<0.002) in media from cells treated with 100 µM of FeCl_3_-NTA than in media from untreated WT controls. Conversely, there was no significant difference between LDH activity in media of hTf Tg-derived MG cells treated with 100 µM FeCl_3_-NTA and that in untreated Tg cell media (Figure 5B).

### Effects of iron stress on mouse and human transferrin expression in Müller glial cells

**Figure 6:**
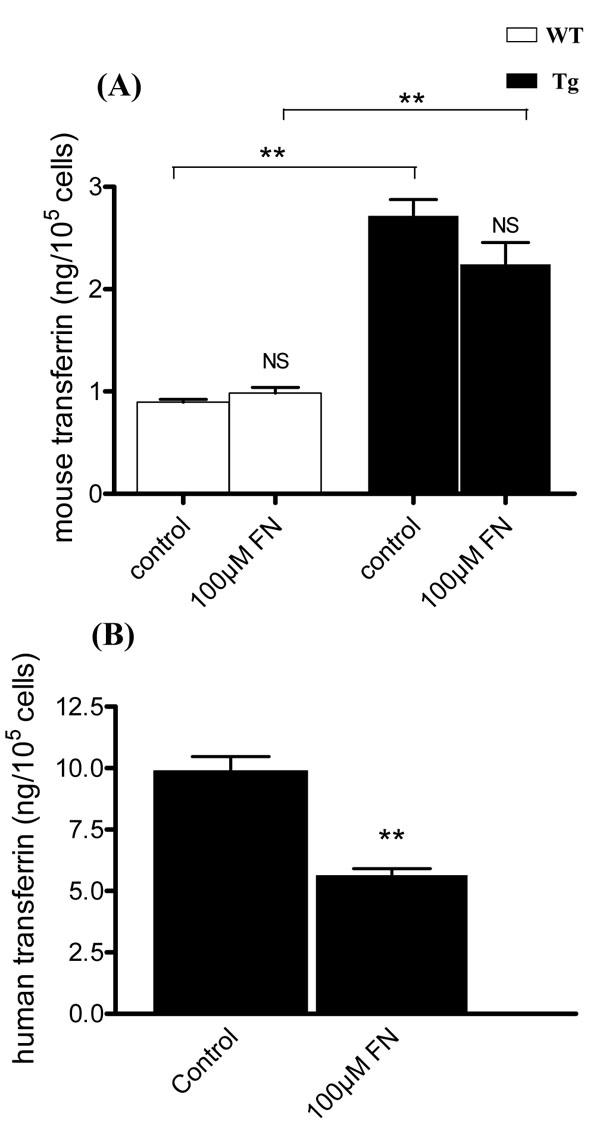
Mouse transferrin and human transferrin proteins expression are modulated after iron stress. A: Mouse transferrin (mTf) secretion was measured by radioimmunoassay (RIA) in the culture medium of Müller glial (MG) cells from wild-type (WT) and transgenic (Tg) mice in control condition or after addition of 100 µM of FeCl_3_-NTA (FN) during 96 h. Each column represents the mean ±SEM. The double asterisk represents statistical significance of differences between treated and control, respectively, for WT and Tg, p<0.01. B: Human transferrin (hTf) secreted was quantified by ELISA assay in the culture medium of MG cells from Tg mice in control condition or after addition of 100 µM of FN during 96 h. Each column represents the mean ±SEM. The double asterisk represents statistical significance of differences from control, p<0.01 In both figures, data considered not significant is marked NS.

MG cell culture media from WT and Tg mice were collected after 96 h of iron stress and mTf and hTf concentrations were measured by RIA and ELISA. The mTf concentration in the medium of untreated Tg-derived MG cells was significantly higher (p<0.002) than in the medium of untreated cells from WT mice (Figure 6A). However, there was no significant difference in mTf concentration between cell culture media treated with and without 100 µM of FeCl_3_-NTA for either WT or Tg mice. Moreover, the hTf concentration in Tg mice MG cell culture media was fivefold higher than mTf concentration. The hTf concentration in the medium of Tg cells treated with 100 µM FeCl_3_-NTA was 50% lower than that in untreated Tg cell medium (Figure 6B). In cell lysates, mTf and hTf were undetectable (data not shown).

**Figure 7:**
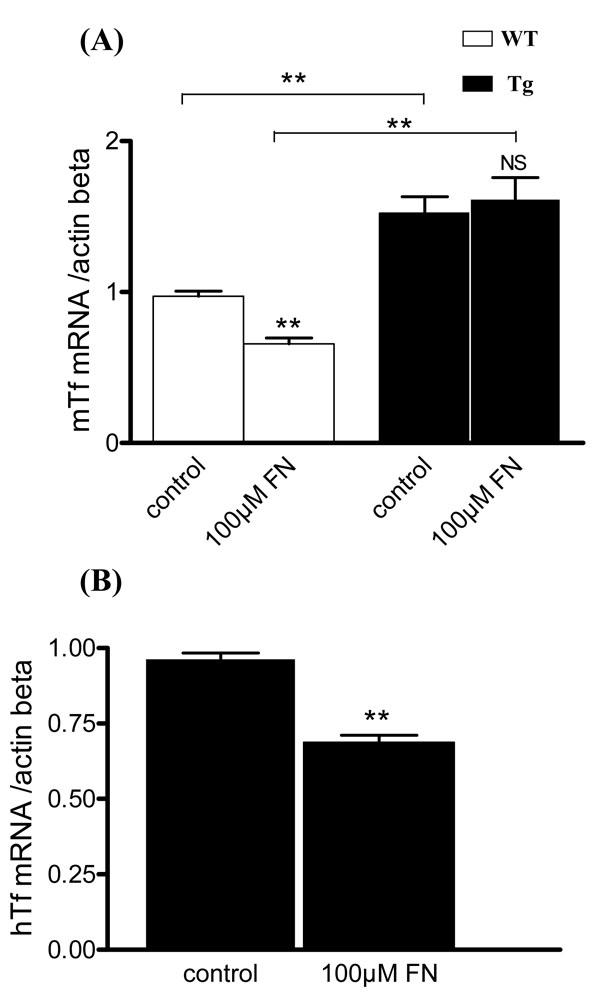
Mouse transferrin and human transferrin mRNA expression are modified after iron stress. A: Mouse transferrin (mTf) mRNA expression was quantified by RT-qPCR in Müller glial (MG) cells respectively from wild-type (WT) and transgenic (Tg) mice in control condition and after addition of 100 µM FeCl_3_-NTA (FN) in the medium during 96 h. Each column represents the mean ± SEM. The double asterisk represents statistical significance of differences between treated and control, respectively, for WT and Tg, and between control and treated, p<0.01. B: Human transferrin (hTf) mRNA expression was quantified by RT-qPCR in MG cells, respectively, from Tg mice in control condition and after addition of 100 µM FN in the medium during 96 h. Each column represents the mean ±SEM. The double asterisk represents statistical significance of differences from control, p<0.01 In both figures, data considered not significant is marked NS.

RT-qPCR was used to determine intracellular mTf and hTf mRNA levels WT and Tg cell cultures following 96 h of iron stress. mTf mRNA was significantly more abundant in MG cells from Tg mice than in those from WT mice, whether treated or untreated (Figure 7A). MG cells from WT mice treated with 100 µM of FeCl_3_-NTA had a significantly (p<0.002) lower level of mTf mRNA expression than untreated WT cells, whereas there was no significant difference between treated and untreated cells from Tg mice. However, Tg cells treated with 100 µM FeCl_3_-NTA had significantly lower (p<0.002) hTf mRNA levels than untreated Tg cells (Figure 7B).

### Addition of exogenous human transferrin in medium of Müller glial cells from wild-type mice after iron stress

**Figure 8:**
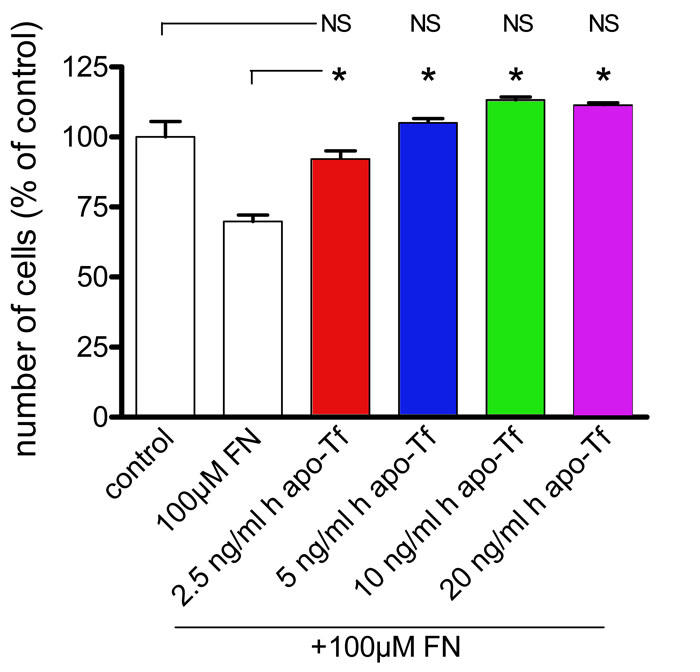
Exogenous human apo-transferrin protects Müller glial cells from wild-type mice from iron-induced stress. The Müller glial (MG) cells from wild-type (WT) mice at the first passage were treated with 100 µM of FeCl_3_-NTA (FN) in presence or absence of different concentrations (2.5, 5, 10, and 20 ng/ml) of human apo-transferrin (h apo-Tf). Their number was determined by counting in comparison to control condition. Each column represents the mean ±SEM. The asterisk represents statistical significance of differences from FN alone, p<0.05. Data considered not significant compared to control are marked NS.

**Figure 9:**
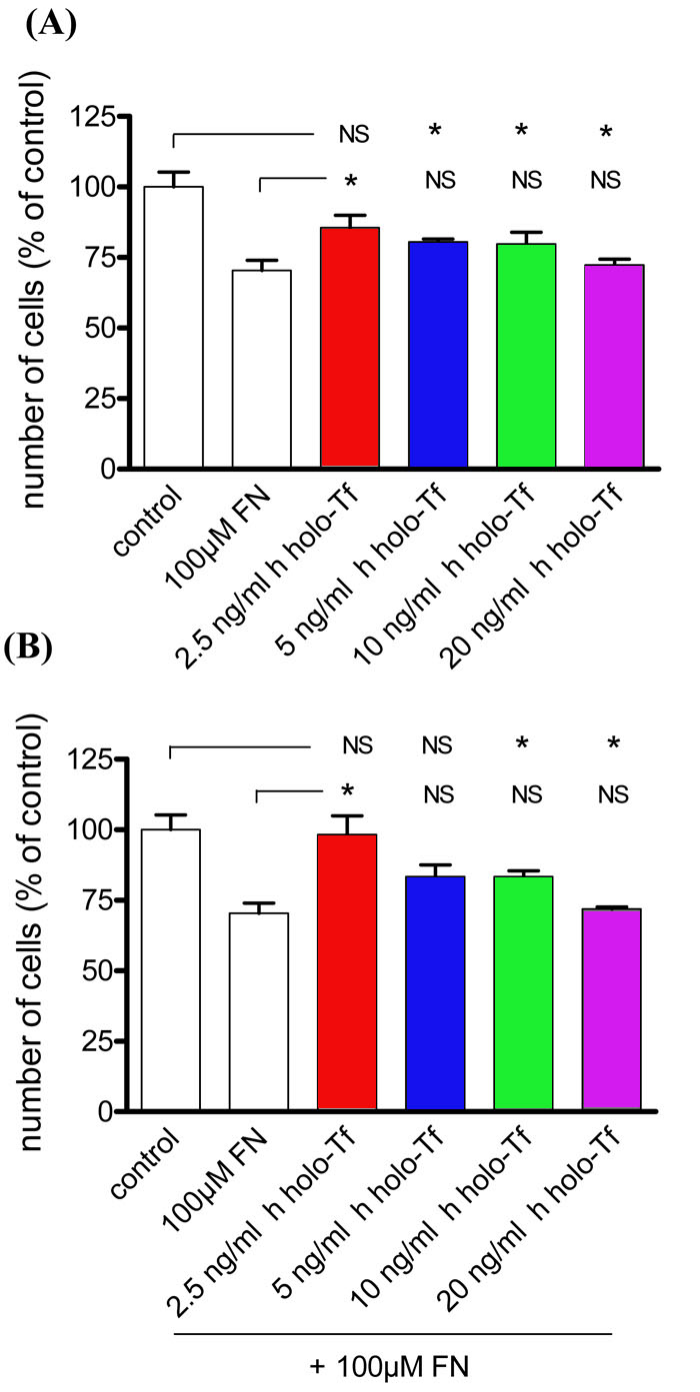
Exogenous human holo-transferrin does not protect Müller glial cells from wild-type mice from iron-induced stress. A,B: The number of Müller glial (MG) cells from wild-type (WT) mice in culture at the first passage after treatment with 100 µM of FeCl_3_-NTA (FN), during 96 h was evaluated by counting in comparison to control condition A: The MG cells from WT mice at the first passage were treated with different concentrations (2.5, 5, 10, and 20 ng/ml) of human holo-transferrin (h holo-Tf). Their number was determined by counting in comparison to control condition. Each column represents the mean ±SEM. The asterisk represents statistical significance of differences for treated by h holo-Tf alone in relation respectively to control and to treated by FN alone, p<0.05 Data considered not significant compared to control or to treated by FN alone is marked NS. B: The MG cells from WT mice at the first passage were treated with 100 µM of FN in presence of different concentrations (2.5, 5, 10, and 20 ng/ml) of h holo-Tf in the medium. Their number was determined by counting in comparison to control condition. Each column represents the mean ±SEM. The asterisk represents statistical significance of differences for treated by h holo-Tf after FN in relation, respectively, to control and to treated by FN alone, p<0.05. Data considered not significant compared to control or to treated by FN alone is marked NS.

We added 2.5, 5, 10, and 20 ng/ml commercial human apo-Tf to WT MG cell culture media containing 100 µM of FeCl_3_-NTA to investigate the potential protective effect of human apo-Tf on WT MG cells. Addition of only exogenous human apo-Tf had no effect on MG cell numbers, even at the highest concentrations (data not shown). However, cells treated with 100 µM of FeCl_3_-NTA and 2.5 ng/ml human apo-Tf had a significantly higher survival rate (92%) than iron-treated cells without addition of exogenous human apo-Tf (70%). Cells with 5, 10, and 20 ng/ml of human apo-Tf added to medium containing 100 µM of FeCl_3_-NTA had survival rates that were 35% to 43% higher than those with FeCl_3_-NTA alone. There was no significant difference between cells with and without exogenous human apo-Tf in the absence of iron (Figure 8). To compare the protective effects of human apo-Tf on WT MG cells, we also added 2.5, 5, 10, and 20 ng/ml commercial human holo-Tf to WT MG cell culture media containing 100 µM of FeCl_3_-NTA. Addition of exogenous human holo-Tf alone at the concentration of 2.5 ng/ml had no significative effect on MG cell numbers. At the highest concentrations (10 and 20 ng/ml), human holo-Tf alone diminished significantly the number of cells compared to condition without iron added (Figure 9A). Cells treated with 100 µM of FeCl_3_-NTA and 2.5 and 5 ng/ml human holo-Tf had a significantly higher survival rate (98 and 83%) than iron-treated cells without addition of exogenous human holo-Tf (70%). Nevertheless, 10 and 20 ng/ml human holo-Tf added to media with 100 µM of FeCl_3_-NTA had no significative effect on survival rate (Figure 9B) compared to iron-treated cells.

## Discussion

Iron is an essential component of cell survival [1], but its capacity to generate highly reactive hydroxyl free radicals via the Fenton reaction can also render it toxic to cells. Iron accumulates in the retina during aging [11,12] and in some retinal degenerative animal models [13,14]. Many proteins such as ferritin, transferrin receptor, and transferrin are involved in regulating iron status. Tf can transport two ferric ions per molecule across the cell membrane, rendering it protective against toxic effects of labile iron. We investigated the regulation of Tf levels in WT and Tg mice. The 16 months-old Tg mice have an apparently normal phenotype, although they have low testicular sperm reserves that do not affect fertility [27]. Retinal structure was not affected and mTf localization was similar in WT and Tg mice in the RPE cell layer, photoreceptor layer, and ganglion cell layer. This same pattern of localization has been described in rat retina [10]. However, in Tg mice, most hTf was in MG and RPE cells, identified using specific markers. This pattern of hTf localization is also observed in human retina; the fact that Tg mice express the hTf gene under the control of its own promoter may explain this similarity [26].

Expression of hTf transgene seemed to affect endogenous mTf production. Indeed, Tg retinal extracts contained significantly more endogenous mTf proteins and mRNA than WT retinal extracts in Tg mice. Similar findings have been reported in Sertoli cells and the brain [25].

We cultured MG cells from Tg and WT mice to determine where Tf is synthesized and transported. MG cells were positively identified in all cultures, using vimentin as a specific marker. hTf immunoreactivity was detected in the cytoplasm of MG cells from Tg mice and in the culture medium. mTf was also detected in the cytoplasm and media of both WT and Tg MG cells. mTf production may be activated in these cells as a stress response caused by culture. In retina from diabetic rats, MG cells upregulate many genes associated with inflammation including Tf [32].

MG cells synthesize and secrete mTf and hTf but lose this ability as the number of passages increase, suggesting that hTf and endogenous mTf promoters have similar activities and are similarly regulated. mTf and hTf promotors both contain the *cis-*acting regulatory elements PRI and PRII, which are similar in the two species [33]. The proximal promoter regions of both hTf and mTf contain a CCAAT consensus sequence that binds to members of the CAAT enhancer-binding protein family of transcription factors [34].

Despite these similarities, there was some discrepancy in the expression of both hTf and mTf in MG cells between Tg and WT mice, which probably corresponds to several differences between hTf and mTf promoter sequences [35]. We observed there was significantly more hTf protein than mTf protein in retinal extract and culture media of MG cells from Tg mice. Thus, mechanisms regulating hTf and mTf are not identical in these Tg mice. Our findings of differences in localization patterns are consistent with this. In the rat brain, Tf mRNA is observed in oligodendrocytes [36] and epithelial cells of the choroid plexus [18]. By contrast, Tf mRNA is not observed in the human choroid plexus [19]. Moreover, hTf transgene is inserted in the F1 area of the chromosome 12 [27] and the number of copies is nearly 12 (F. Guillou, personal communication), which could explain the specificity of expression noticed.

Tf has many functions; most are related to its Fe^3+^-binding and -transporting properties, rendering it a potent antioxidant. In renal ischemia-reperfusion injury, Tf lowers the circulating redox-active iron levels [37]. Tf has potent anti-apoptotic/cytoprotective effects against Fas-mediated signals in hepatocytes and lymphohemopoietic cells [38,39]. In a rabbit model of after-cataract formation following cataract surgery, Tf synthesis is upregulated in lens epithelia acting as a survival and proliferative factor [40]. These properties can be used to prevent iron accumulation and free radical formation by the Fenton reaction in many situations [37]. For example, ovoTf is a survival and growth-promoting factor [41]. Tf may function as a neurotransmitter or neuromodulator in the developing vertebrate nervous system [42]. In addition, MG cells mediate nerve cell protection [43–45]. Moreover, MG cells are potential regenerative stem/precursor cells involved in neural retina regeneration response in vertebrates following lesion [46,47].

We subjected these cells to iron-induced stress using NTA, increasing levels of bound and unbound iron in the cytoplasm [46–48] without activation of the classical Tf receptor endocytosis pathway [49,50].

We observed that treatment with 100 µM FeCl_3_-NTA did not affect survival of Tg MG cells during the first passage, whereas there were 35% fewer cells in WT cultures following treatment with 100 µM FeCl_3_-NTA than there were in untreated WT cultures. During the third passage of Tg MG cells, hTf could no longer be detected in the medium and the effects of iron treatment were similar in cells from both WT and Tg mice. Cell survival correlated with hTf concentration during the subculture. Moreover, WT MG cells treated with human apo-Tf but not human holo-Tf were protected against 100 µM FeCl_3_-NTA. Unlike human holo-Tf, WT MG cells survival against iron treatment is correlated to the capacity of human apo-Tf to catch iron in excess. Human holo-Tf, which probably is not fully saturated with iron, seemed to be protective at the lowest concentrations (2.5 and 5 ng/ml). At the highest concentrations (10 and 20 ng/ml), it seemed to be toxic because its iron loads were higher.

We quantified the levels of Tf protein and mRNA in serum-free medium to assess the effects of iron-induced stress on hTf and mTf metabolism in MG cells. As expected mRNA levels were slightly lower for mTf and markedly lower for hTf in the presence than absence of iron. The molecular basis for the lower levels of hTf following iron administration has not been extensively studied. In humans, anemia resulting in iron deficiency is associated with high plasma Tf levels whereas conditions resulting in excess iron stores are associated with low plasma Tf [51]. The low plasma Tf concentration associated with increased iron stores in humans may be due to the effects of negative feedback on Tf synthesis [52].

Tf gene transcription is induced by a variety of factors in a tissue-specific manner. In rats and chickens, nutritional iron deficiency activates Tf gene transcription specifically in the liver, resulting in a several-fold increase in Tf synthesis [53,54]. In human hepatoma HepG2 cells lines, iron treatment suppresses Tf synthesis demonstrating regulation of intact endogenous Tf synthesis by iron [54]. Previous studies on transgenic mice have identified a 50 nucleotide region in the 5′UTR of hTf mRNA involved in hTf iron regulation. Within this region, an an iron responsive element (IRE)- homologous sequence (“the putative hTf IRE”) was identified [55].

The high level of hTf in the medium of hTf Tg MG cells may be a protective mechanism against the labile iron pool. Tf sequestrates iron from the FeCl_3_-NTA added to the medium, preventing iron toxicity. The use of FeCl_3_-NTA permits direct entry of iron into the MG cells where it is stored by ferritin. Iron excess inhibits activation of Tf transcription. Very low levels of hTf mRNA may result from transcription factors binding specific hTf promoter sequences. hTf can regulate its transcription directly or indirectly in an autocrine manner. hTf is an autocrine factor for the development and maturation of oligodendrocytes [56]. In humans, Tf produced by T cells can function in an autocrine manner rather than a paracrine manner [57]. Tf synthesis in small cell lung cancer cells also acts as an autocrine regulator of cellular proliferation, allowing these cells to continue proliferating under transferrin-free conditions [58].

In our study, isolated MG cells from Tg mice expressing hTf were protected against iron-mediated stress. We believe that hTf regulates its transcription in an autocrine manner to control intracellular iron levels. These findings are consistent with a potential protective role for Tf in retina, which is related to the capacity of Tf to bind iron. Thus, therapeutic targeting of retinal Tf may be beneficial in pathological conditions involving iron accumulation such as aging, AMD, and inflammation.
